# Supplementary material for: Universal Preparation Strategy for Ultradurable Antibacterial Fabrics through Coating an Adhesive Nanosilver Glue
Source: Nanomaterials (Basel). 2022 Jul 15;12(14):2429. doi: 10.3390/nano12142429 (PMC9323275; doi:10.3390/nano12142429)
Supplement: Supplementary file 1 [file nanomaterials-12-02429-s001.zip › nanomaterials-1791802-SI.pdf]

nanomaterials

Article

Universal Preparation Strategy for Ultradurable Antibacterial Fabrics through Coating an Adhesive Nanosilver Glue

Jundan Feng [1,†], Lingling Feng [1,†], Sijun Xu [1,*], Chunhong Zhu [2], Gangwei Pan [1] and Lirong Yao [1]

[1] National & Local Joint Engineering Research Center of Technical Fiber Composites for Safety and Protection, Nantong University, Nantong 226019, China; feng5017@hotmail.com (J.F.); Feng20220606@hotmail.com (L.F.); pangangwei@ntu.edu.cn (G.P.); ylr8231@ntu.edu.cn (L.Y.)
[2] Faculty of Textile Science and Technology, Shinshu University, Nagano 386-8567, Japan; zhu@shinshu-u.ac.jp
* Correspondence: xusijunwork@hotmail.com
† These authors contributed equally to the paper.

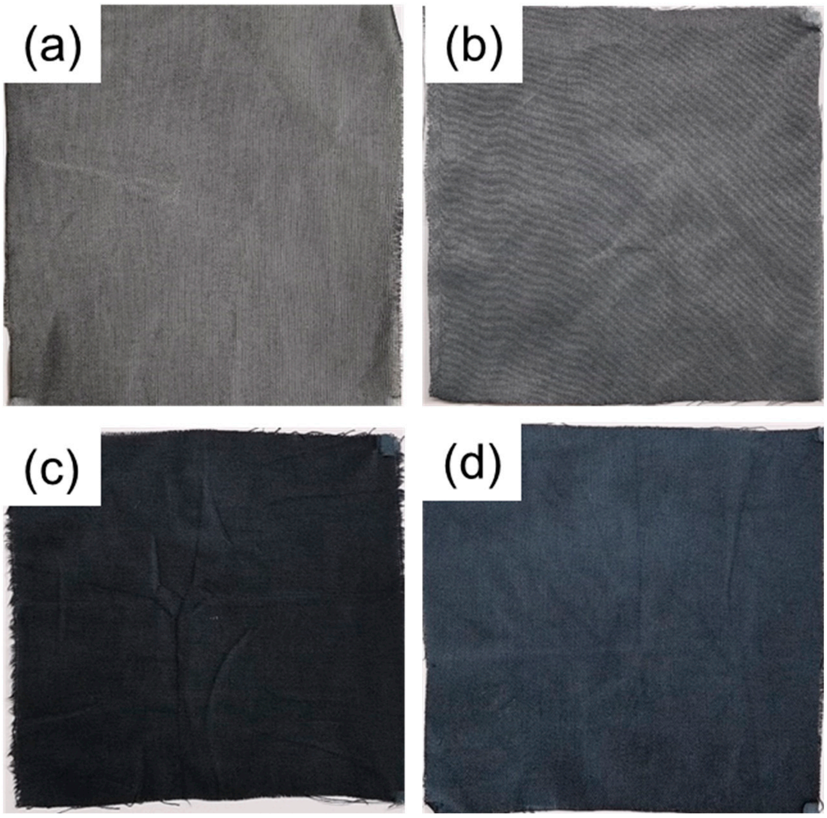

Figure S1. Photos of cotton fabrics dyed with the (a) pure nanosilver glue and (b) mixture of nanosilver glue and reactive black KN-B and polyester fabrics dyed with the (c) pure nanosilver glue and (d) mixture of nanosilver glue and reactive black KN-B.

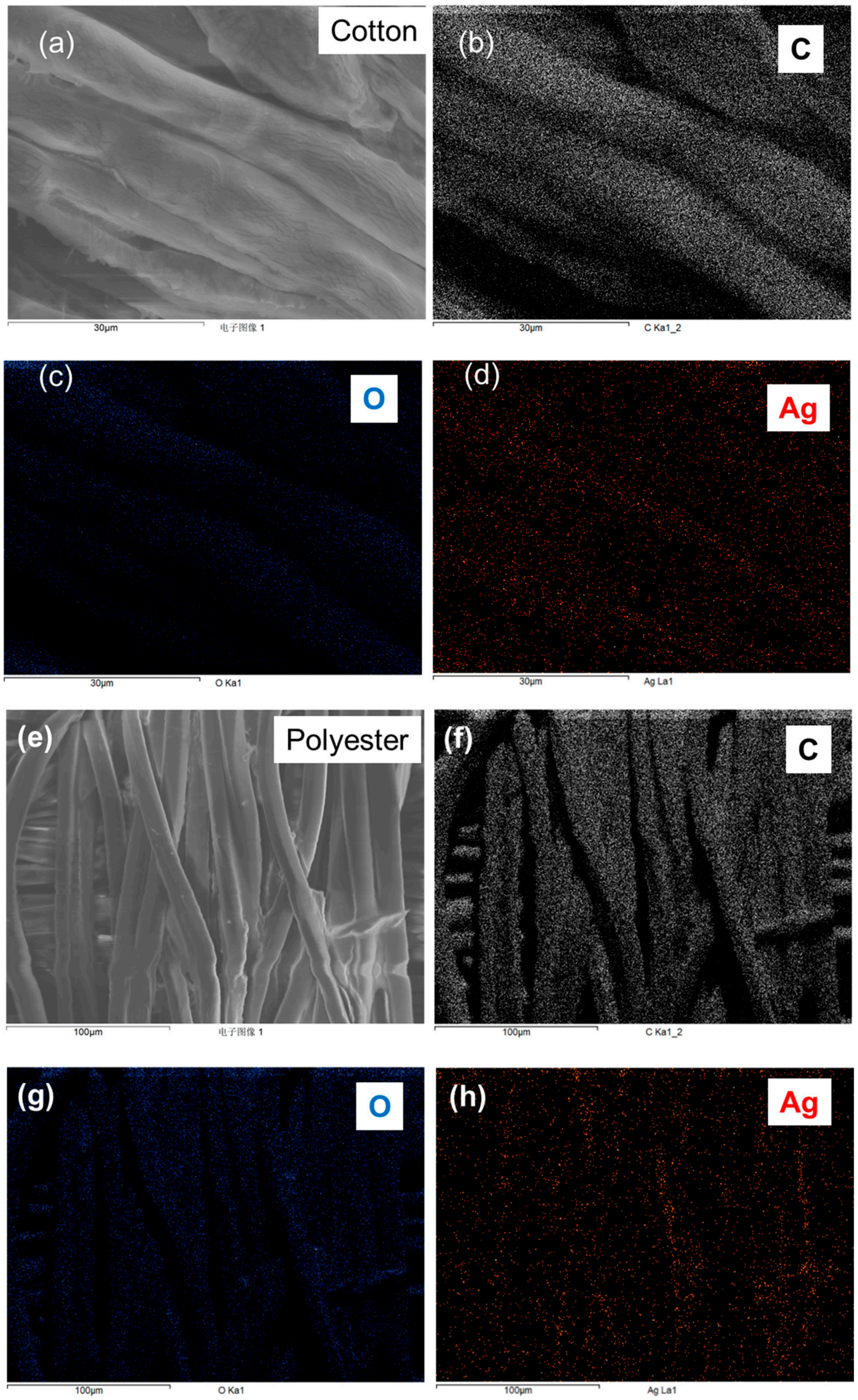

Figure S2. EDS maps of (a-d) treated cotton and (e-h) polyester fabrics.

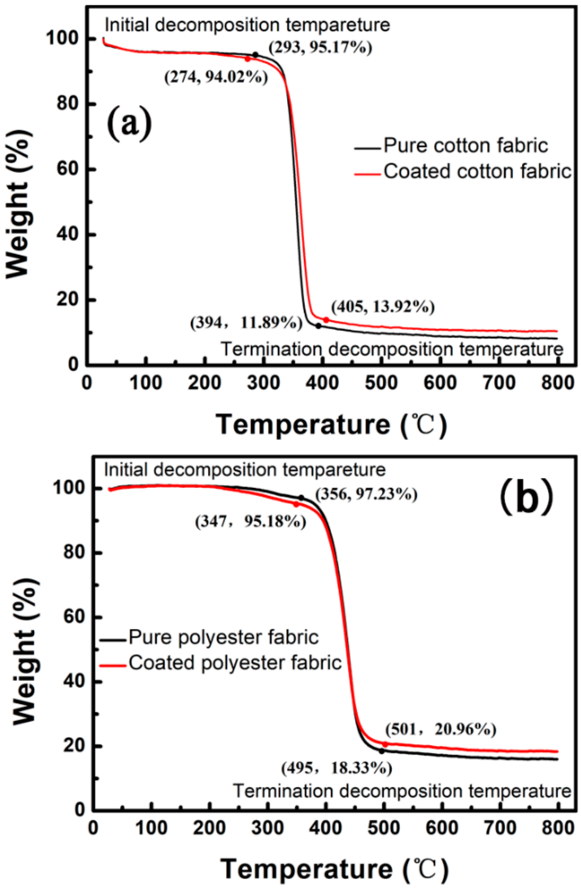

Figure S3. Thermogravimetric (TG) curves of (a) cotton and (b) polyester fabric samples.

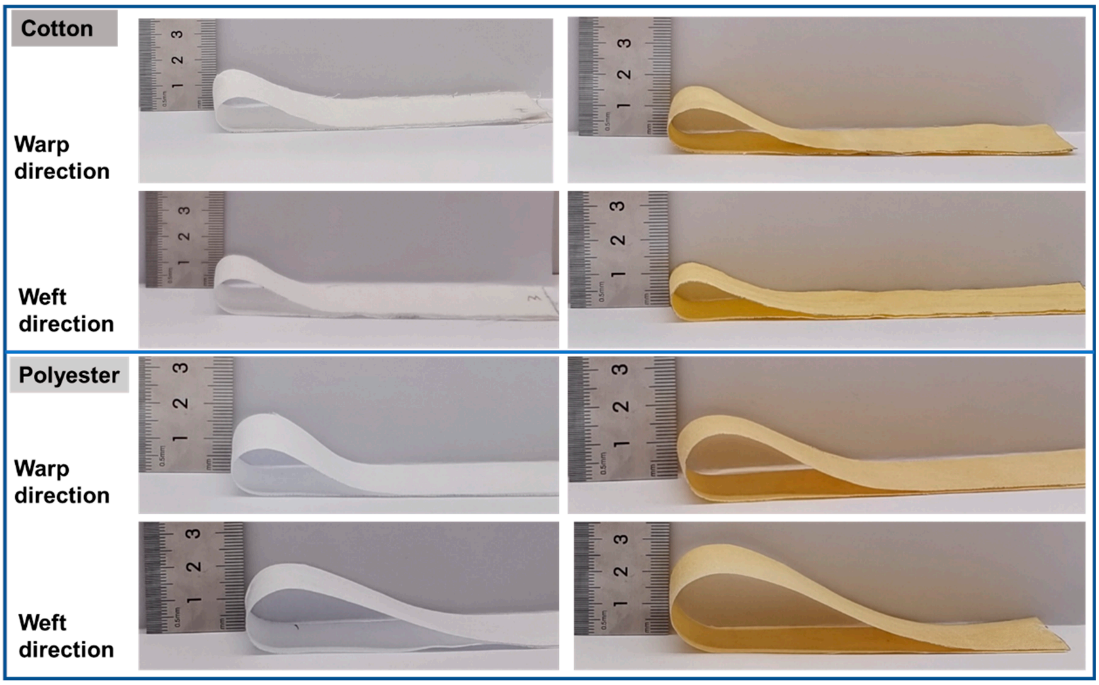

Figure S4. The flexibility of (left) untreated and (right) treated cotton and polyester fabrics in the warp and weft directions.
